# Graph pangenome reveals the regulation of malate content in blood-fleshed peach by NAC transcription factors

**DOI:** 10.1186/s13059-024-03470-w

**Published:** 2025-01-09

**Authors:** Wenbo Chen, Qi Xie, Jia Fu, Shaojia Li, Yanna Shi, Jiao Lu, Yuanyuan Zhang, Yingjie Zhao, Ruijuan Ma, Baijun Li, Bo Zhang, Donald Grierson, Mingliang Yu, Zhangjun Fei, Kunsong Chen

**Affiliations:** 1 College of Agriculture & Biotechnology, Zhejiang University, Hangzhou, 310058 China; 2 Zhejiang Key Laboratory of Horticultural Crop Quality Improvement, Zhejiang University, Hangzhou, 310058 China; 3 The State Agriculture Ministry Laboratory of Horticultural Plant Growth, Development and Quality Improvement, Zhejiang University, Hangzhou, 310058 China; 4 Institute of Pomology, Jiangsu Key Laboratory for Horticultural Crop Genetic Improvement, Jiangsu Academy of Agricultural Sciences, Nanjing, 210014 China; 5 Division of Plant and Crop Sciences, School of Biosciences, University of Nottingham, Loughborough, LE125RD UK; 6 Boyce Thompson Institute, Cornell University, Ithaca, 14853 USA; 7 USDA-ARS Robert W. Holley Center for Agriculture and Health, Ithaca, 14853 USA

**Keywords:** Graph pangenome, Peach, Malate content, Blood-fleshed, NAC

## Abstract

**Background:**

Fruit acidity and color are important quality attributes in peaches. Although there are some exceptions, blood-fleshed peaches typically have a sour taste. However, little is known about the genetic variations linking organic acid and color regulation in peaches.

**Results:**

Here, we report a peach graph-based pangenome constructed from sixteen individual genome assemblies, capturing abundant structural variations and 82.3 Mb of sequences absent in the reference genome. Pangenome analysis reveals a long terminal repeat retrotransposon insertion in the promoter of the NAC transcription factor (TF) *PpBL* in blood-fleshed peaches, which enhances *PpBL* expression*.* Genome-wide association study identifies a significant association between *PpBL* and malate content. Silencing *PpBL* in peach fruit and ectopic overexpression of *PpBL* in tomatoes confirm that *PpBL* is a positive regulator of malate accumulation. Furthermore, we demonstrate that PpBL works synergistically with another NAC TF, PpNAC1, to activate the transcription of the aluminum-activated malate transporter *PpALMT4*, leading to increased malate content.

**Conclusions:**

These findings, along with previous research showing that PpBL and PpNAC1 also regulate anthocyanin accumulation, explain the red coloration and sour taste in blood-fleshed peach fruits.

**Supplementary Information:**

The online version contains supplementary material available at 10.1186/s13059-024-03470-w.

## Background

Peach (*Prunus persica*), a member of the Rosaceae family, is one of the most economically important fruit crops. Peach cultivars can be classified into three groups based on flesh color: white-, yellow-, and blood-fleshed. In China, both yellow- and blood-fleshed peach cultivars have a long history of selection and cultivation [1]. The red coloration in blood-fleshed peaches is due to anthocyanins, water-soluble compounds responsible for red, purple, and blue colors in flowers, fruits, vegetables, seeds, and storage organs [2–7]. Anthocyanins not only play an important role in attracting animals for pollination and seed dispersal, but also have numerous health benefits, such as antioxidant and anti-inflammatory properties [8, 9]. The regulation of anthocyanin biosynthesis in fruit is a complex process that involves the interaction of multiple genes and environmental factors. Several studies have investigated the molecular mechanisms underlying anthocyanin accumulation in various peach tissues, including fruit skin [10, 11], endocarps [12], leaves [13], and flowers [14]. Zhou et al. [15] reported that a NAC transcription factor (TF), designated as BLOOD (BL), shows high expression levels in blood-fleshed peach fruit at late developmental stages and is the key gene responsible for the blood-fleshed trait in peach. PpBL forms a heterodimer with PpNAC1 to activate the expression of *PpMYB10.1*, a major regulator of anthocyanin accumulation in peach fruit [16].

Blood-fleshed peaches typically have a slightly more intense sour flavor compared to traditional peaches. Acidity is an important determinant of the overall organoleptic quality of most fruits and is mainly determined by the accumulation of various organic acids, such as malate, citrate, quinate, and tartrate, with malate and citrate being the most common [17]. The accumulation of organic acids in the vacuole, which is also the site of anthocyanin accumulation, during fruit development and ripening is governed by several processes, including synthesis, degradation, and transport [17]. Recently, several genes involved in vacuolar organic acid transport have been identified as critical regulators of fruit acidity. For example, *Ma1* in apple and *Sl-ALMT9* in tomato, which encode an aluminum-activated malate transporter (ALMT), control fruit acidity [18, 19]. Similarly, transient overexpression of *PpALMT1* promotes malate accumulation in peach flesh [20]. Genome-wide association studies (GWAS) and comparative transcriptome analyses have identified *PpTST1*, encoding a tonoplast sugar transporter, as a strong candidate gene for organic acid accumulation. Overexpression of *PpTST1*^His^ reduces organic acid content in both peach and tomato fruits [21]. GWAS analysis of citrate content, combined with transient expression assays, has shown that *Prupe.5G006500*, which encodes a V-type proton ATPase subunit F, positively affects citrate, malate, and quinate levels in peach fruit [22]. However, to date, most studies on organic acid in peach fruit have focused on structural genes, with little known about upstream factors, such as TFs, involved in regulating fruit organic acid accumulation.

Genomics and population genetics are powerful tools for studying the genetic architecture underlying various traits crucial for peach cultivation. A major milestone in peach genomics was the successful sequencing of the peach reference genome [23], which has enabled comprehensive functional annotation and facilitated the identification and characterization of key genes governing important biological processes in peaches. Previous genomic studies have elucidated the genetic basis of important agronomic traits, such as fruit ripening and aroma synthesis [20, 24–26]. Population-level analyses have uncovered natural genetic variations among different peach cultivars and wild relatives, providing valuable insights into the evolutionary and domestication history of peach [27]. Recently, the advent of pangenomics has ushered in a transformative era in plant research, offering a comprehensive view of genetic diversity and evolutionary dynamics within species. Since the first plant pangenome analysis in soybean [28], pangenome research has been extended to many other plant species, including Arabidopsis, rice, maize, barley, cotton, tomato, potato, cabbage, mung bean, citrus, and pear [29–40], highlighting the great potential of pangenomes for functional genomic discoveries.

In this study, we have assembled high-quality genomes of “Hu Jing Mi Lu” (HJML) and “Feng Hua Yu Lu” (FHYL), two improved Chinese peach cultivars with white flesh, a low-acid taste, and distinct aroma. We then construct a peach graph-based pangenome from the genome assemblies of 16 cultivated peaches. Analysis of structural variants (SVs) captured in the graph pangenome reveals an LTR retrotransposon in the promoter of the NAC TF *PpBL* that is present only in blood-fleshed peaches and increases the transcript levels of *PpBL*. Our GWAS suggests that *PpBL* is strongly associated with malate accumulation in peach fruit. Gene-silencing and transient overexpression confirm that PpBL works synergistically with PpNAC1, increasing malate content by up-regulating the aluminum-activated malate transporter *PpALMT4.* A previous study [15] has demonstrated that PpBL regulates anthocyanin accumulation in peach fruit through activating *PpMYB10.1*. Together, these findings provide valuable insights into the regulation of both anthocyanin and malate accumulation in blood-fleshed peach fruits by NAC TFs.

## Results

### Graph pangenome of cultivated peach

We first assembled chromosome-level high-quality genomes for two peach cultivars, HJML and FHYL. We generated a total of 108 × coverage of PacBio HiFi, 317 × coverage of Hi-C and 108 × coverage of Illumina sequences for HJML and 106 × coverage of PacBio HiFi, 103 × coverage of Hi-C and 98 × coverage of Illumina sequences for FHYL (Additional file 1: Table S1). The PacBio HiFi reads were de novo assembled into contigs, which were subsequently clustered into eight pseudo-chromosomes using Hi-C reads. The total assembly sizes were 221.2 Mb for HJML and 232.3 Mb for FHYL (Additional file 1: Table S2). The N50 contig sizes were 7.0 Mb for HJML and 4.1 Mb for FHYL (Additional file 1: Table S2). Comprehensive evaluations confirmed the high quality of both genome assemblies (Additional file 1: Table S3). A total of 91.0 Mb (41.2%) and 98.9 Mb (42.6%) of repeat sequences were identified in the HJML and FHYL genomes, respectively, slightly lower than that in the Lovell v2.0 genome (44.3%) (Additional file 1: Table S4). The most abundant repeats were annotated as long terminal repeats (LTRs), accounting for 17.2% of the genome assemblies. A total of 27,926 protein-coding genes were predicted in the HJML genome and 28,972 in the FHYL genome.

**Fig. 1:**
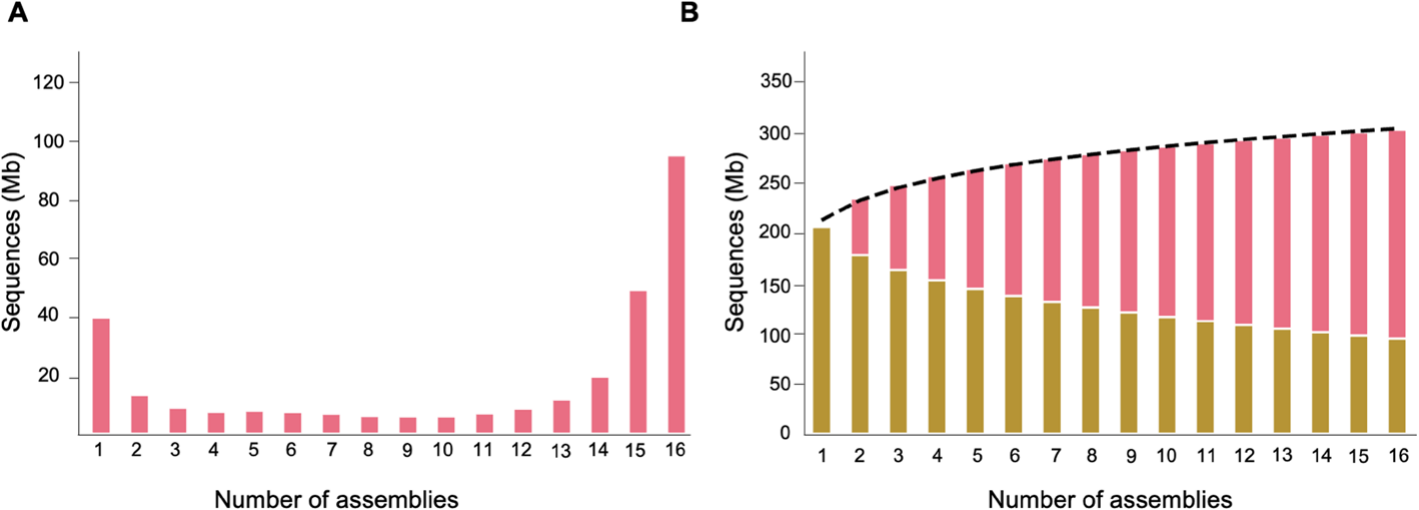
Peach graph-based pangenome. **A** Total sequences shared by varying numbers of genome assemblies in the peach graph pangenome. **B** Simulation of the increase in pan-genome size and the decrease of core-genome size, calculated using Panacus (https://github.com/marschall-lab/panacus). Red bars indicate sequences present in at least one of the varying numbers of genome assemblies (pangenome), while yellow bars indicate sequences present in all the varying numbers of genome assemblies (core genome)

Using these two newly generated assemblies, along with published genome assemblies from 14 cultivated peach accessions (Additional file 1: Table S5), we constructed a graph-based pangenome of cultivated peach using the Minigraph-Cactus package [41], which takes multiple assemblies as input, performs whole-genome alignments, and derives a pangenome graph from these alignments. The 16 assemblies used in this study represent a diverse set of domesticated peaches, exhibiting significant variation in fruit size, fruit shape, flesh color, fruit texture, flavor, and aroma (Additional file 1: Table S5; Additional file 2: Fig. S1). Using the HJML assembly as the backbone, Minigraph-Cactus progressively added complexity to the graph by aligning the other genome assemblies to the HJML genome. Unaligned (non-reference) sequences were represented as distinct paths composed of sequence nodes. The resulting pangenome graph contained 10,911,706 nodes and 14,925,688 edges, yielding a mean degree, or average number of edges per node, of 1.37. The total length of sequences represented in the graph was 303,531,492 bp, with 82.3 Mb of sequences absent from the HJML assembly. A total of 39.9 Mb of sequences in the graph pangenome were unique to a single accession, while 144.6 Mb of sequences were present in 15 or 16 assemblies, representing the core genome (Fig. 1A). We then constructed a gene-based pangenome from the 16 genome assemblies, comprising a total of 34,536 orthologous gene groups. Genes in the pangenome were categorized into core (present in 15 or 16 genomes; 15,938 genes, 46.2%), softcore (present in 14 genomes; 1964, 5.7%), dispensable (present in 2–13 genomes; 12,556, 36.4%), and private (present in only one genome; 4078, 11.8%) (Additional file 2: Fig. S2A). Gene ontology (GO) analysis revealed that dispensable and private genes were significantly enriched with those involved in biological processes such as defense response, response to stimulus, and signal transduction, while core genes were significantly enriched in biological processes related to conserved and essential functions, including oxidation–reduction process, regulation of RNA biosynthetic process, cellular biosynthetic process (Additional file 1: Table S6). Modeling of pangenome sizes suggested a closed/saturated pangenome for cultivated peach (Fig. 1B, Additional file 2: Fig. S2B).

As the 16 peach genome assemblies were encoded as paths in the pangenome graph, we characterized variants through graph decomposition. We identified 2,078,553 small variants (< 50 bp) and 48,444 large SVs (> 50 bp) within the graph. The average length of SVs in the peach graph pangenome was 4244 bp (Additional file 2: Fig. S3). These SVs were unevenly distributed across the peach chromosomes, with certain regions exhibiting a high density of SVs (Additional file 2: Fig. S4). The majority of SVs (37,533 of 48,444; 77.5%) were located in intergenic regions (Additional file 1: Table S7), with 35.2% (13,200) positioned within 2 kb upstream of 9007 protein-coding genes. Only 4636 (9.6%) SVs were located in exons, affecting a total of 3191 genes. GO analysis indicated that these genes were enriched in biological processes such as DNA metabolic process, signaling, xenobiotic transport, and cellular response to stimulus, which may be linked to peach adaptations to various local environments (Additional file 1: Table S8).

### SV genotyping in cultivated peach population

**Fig. 2:**
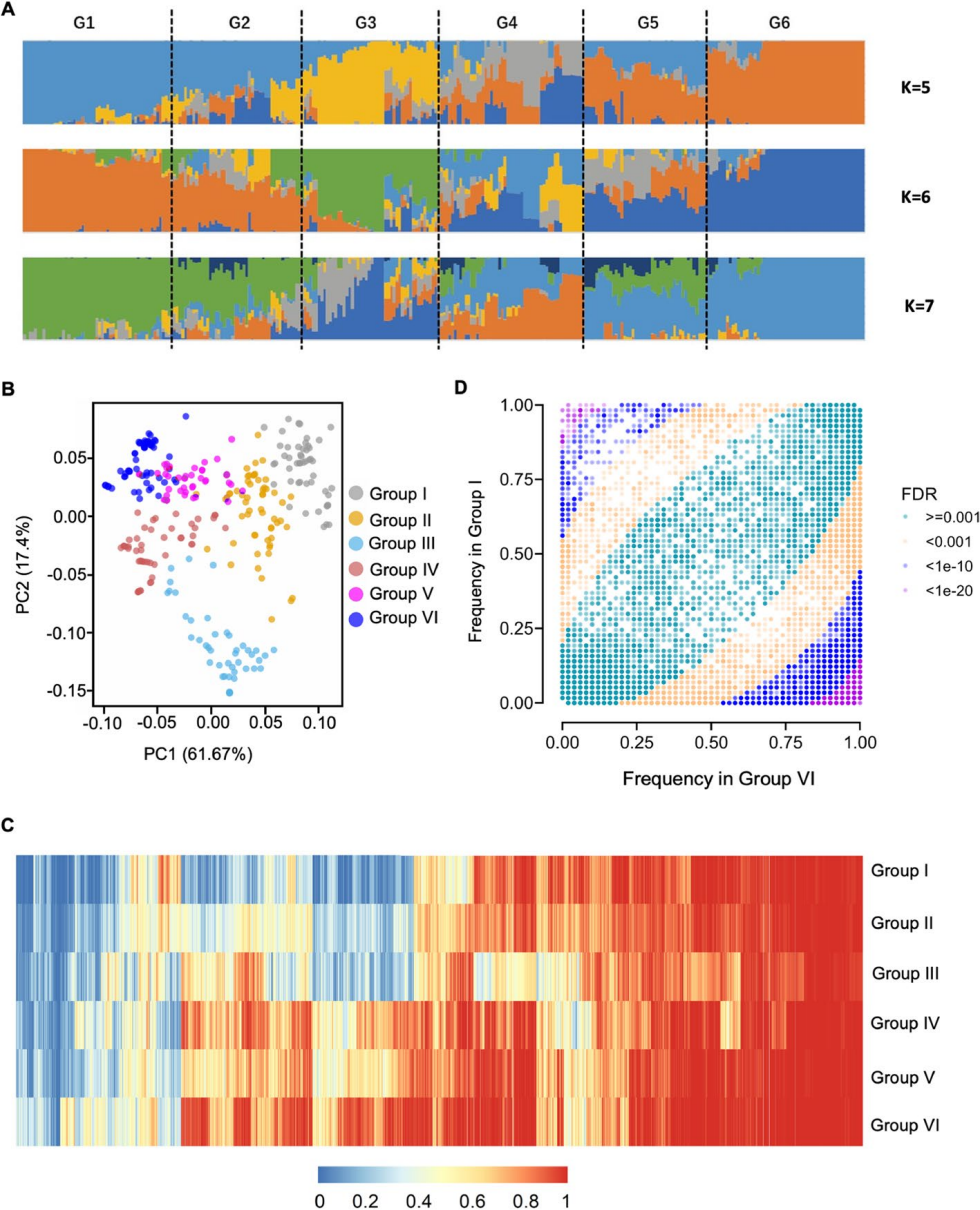
SVs in six peach groups.** A** Population structure of 301 peach accessions. **B** PCA of 301 peach accessions. **C** Reference allele frequency patterns of SVs across the six peach groups. **D** Scatter plots comparing reference allele frequencies of SVs between Group I and Group VI

We performed genome resequencing on 301 peach cultivars from various geographical regions, achieving an average sequencing depth of approximately 25.6 × (Additional file 1: Table S9). Using the HJLM genome assembly as the reference, we identified a total of 741,406 high-quality SNPs and small indels across the 301 peach accessions, with a minor allele frequency (MAF) ≥ 0.05 and a missing data rate < 20%. Population structure analysis based on these variants divided these accessions into six groups (Fig. 2A) according to the best *K* = 6 (Additional file 2: Fig. S5) and principal component analysis (PCA) (Fig. 2B). Group I mainly comprised western cultivars, notably from the Americas and Europe (Additional file 1: Table S9). Group II contained a mixture of both western and eastern cultivars, while Group III consisted predominantly of landraces from eastern countries. Groups IV to VI were largely composed of improved eastern cultivars. We measured the fruit malate content and firmness of these 301 peach cultivars (Additional file 2: Fig. S6). Group I showed higher fruit malate content and fruit firmness than Group VI, indicating divergent selection for fruit acidity and firmness between western and eastern peach cultivars.

We then applied PanGenie [42] to genotype SVs captured in the graph pangenome for the 301 peach accessions using short sequencing reads. The reference allele frequencies of these SVs showed distinct patterns across the six peach groups (Fig. 2C). We identified 3143 SVs with significantly higher reference allele frequencies in Group VI compared to Group I (Fig. 2D), with 771 and 189 located in the upstream regions (2 kb) and exons of protein-coding genes, respectively (Additional file 1: Tables S10 and S11). Notably, six genes encoding pectin lyase-like superfamily proteins (*Hjml.2g002955*, *Hjml.4g000419*, *Hjml.4g000976*, *Hjml.7g002037*, *Hjml.7g002390*, *Hjml.7g002392*) and three genes encoding pectinesterase (*Hjml.2g002303*, *Hjml.2g002921*, *Hjml.2g002922*) contained SVs in their upstream regions, potentially explaining the different fruit textures between Group I and Group VI accessions (Additional file 2: Fig. S6). We also identified 515 SVs with higher reference allele frequencies in Group I compared to Group VI, with 192 and 44 located in the upstream regions and exons of genes, respectively (Additional file 1: Tables S12 and S13). A gene encoding the aluminum-activated malate transporter 2 (*Hjml.6g001497*) and two genes encoding NB-ARC domain-containing disease resistance proteins (*Hjml.1g000455*, *Hjml.1g002781*) contained SVs in their upstream regions. These findings provide insights into the improved fruit quality and reduced disease resistance observed in modern improved peach cultivars (Group VI) during domestication.

### A retrotransposon insertion in the promoter of *PpBL* enhances its expression

**Fig. 3:**
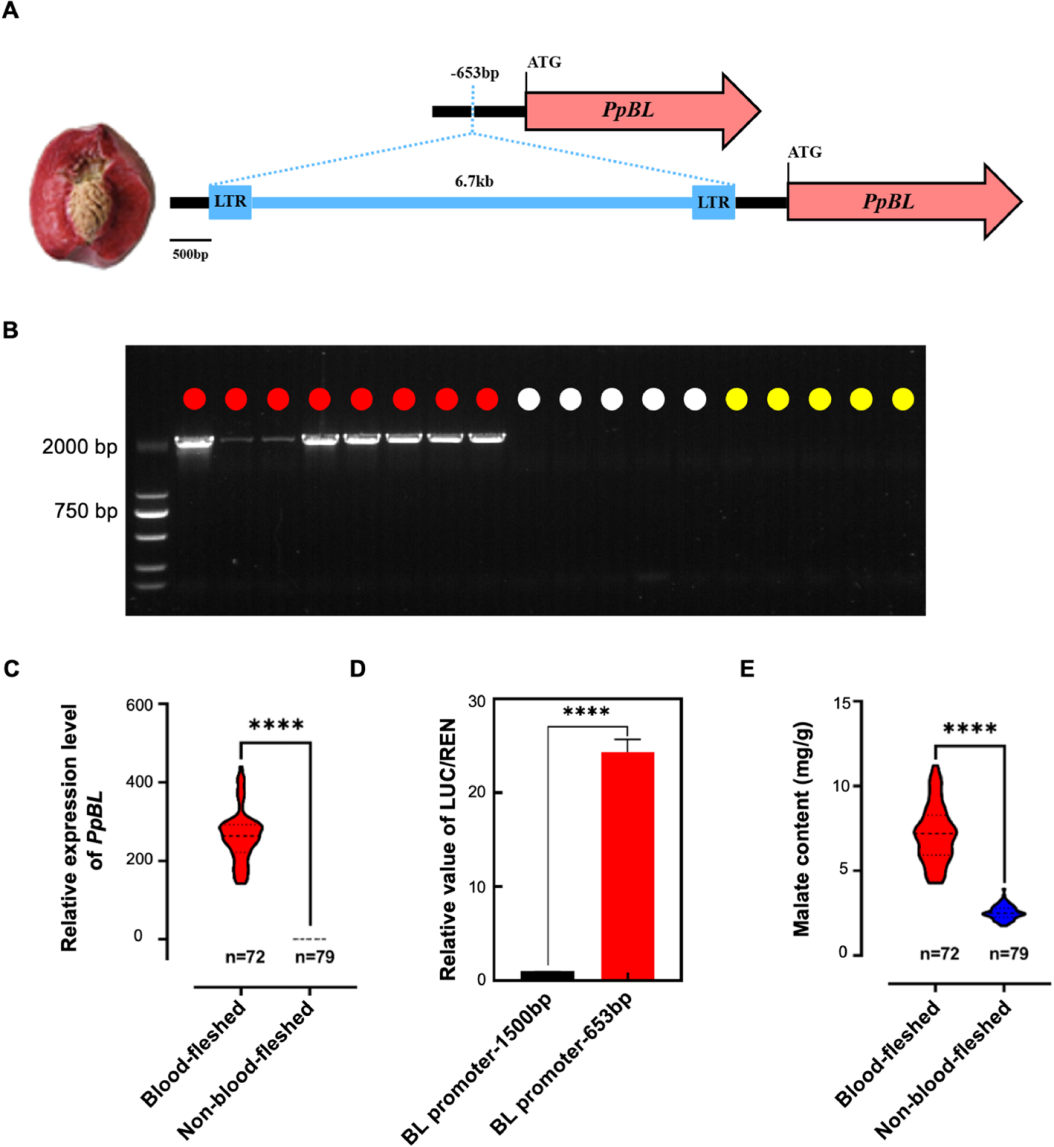
A retrotransposon insertion in the promoter of *PpBL* enhances its expression in blood-fleshed peaches.** A** Graphic representation of the 6.7-kb LTR retrotransposon insertion in the *PpBL* promoter in blood-fleshed peaches. **B** PCR validation of the LTR retrotransposon insertion in 18 peaches. Red, white, and yellow circles mark the blood-, white-, and yellow-fleshed peach accessions, respectively. **C** Expression (FPKM values) of *PpBL* in blood-fleshed (*n* = 72) and non-blood-fleshed (*n* = 79) peaches in the F_1_ population of the blood-fleshed peach cv. C25-12–11 and the yellow-fleshed cv. Frederick. **D** Dual-luciferase assays of the ability of the two *PpBL* promoter fragments in activating gene transcription. The LUC/REN ratio of the 1500-bp *PpBL* promoter fragment (BL promoter-1500 bp) was used as the calibrator (set at a value of 1). Error bars represent the SE from three biological replicates. **E** Malate content in blood-fleshed (*n* = 72) and non-blood-fleshed (*n* = 79) peaches in the F_1_ population of cv. C25-12–11 and cv. Frederick. Asterisks indicate significant differences (∗ ∗  ∗  ∗ *P* < 0.0001, two-tailed Student’s *t*-test)

Our SV analysis in the graph pangenome identified a 6.7-kb LTR retrotransposon insertion located 653 bp upstream of the start codon of *PpBL* (*Hjml.5g000099*) in the genome of the blood-fleshed peach cv. “Tian Jin Shui Mi,” one of the sixteen genomes used for graph pangenome construction (Fig. 3A). *PpBL* has been reported to be highly upregulated in the blood-fleshed peach cultivar “Da Hong Pao,” and virus-induced gene silencing of *PpBL* has been shown to reduce anthocyanin pigmentation [15]. The presence of this insertion in the eight blood-fleshed peaches and its absence in the ten non-blood-fleshed peaches were validated by PCR (Fig. 3B). The LTR insertion was found in all 15 blood-fleshed peaches within the 301-peach collection, as confirmed by genome resequencing read mapping (Additional file 2: Fig. S7). An F_1_ population derived from the cross between the blood-fleshed peach cv. C25-12–11 and the yellow-fleshed cv. Frederick was constructed, which comprised 151 progenies, of which 72 were blood-fleshed and 79 were non-blood-fleshed (Additional file 2: Fig. S8). The expression of *PpBL* in the 72 blood-fleshed progenies was significantly higher than in the non-blood-fleshed progenies (Fig. 3C and Additional file 1: Table S14). Two genome fragments, 653 bp and 1500 bp upstream of the start codon of cultivar HJML, were cloned into the pGreen II 0800-LUC vector, to compare their activation activities. Results from the dual-luciferase assay indicated that the activation activity of the 653-bp promoter fragment was much higher than that of the 1500-bp fragment (Fig. 3D), suggesting that the LTR insertion may disrupt the binding of repressors to the promoter of *PpBL*, thereby enhancing its expression.

### *PpBL* promotes malate accumulation in peach

With some exceptions, blood-fleshed peaches usually have a sour taste. Malate is the most abundant organic acid in peach fruit [43]. We measured the malate content at different fruit developmental stages of the white-fleshed peach “XHH” and the blood-fleshed peach “TJSM,” revealing that “TJSM” had a higher malate content than “XHH” (Additional file 2: Fig. S9). We further measured the malate contents in the F_1_ population derived from the cross between the blood-fleshed peach cv. C25-12–11 and the yellow-fleshed cv. Frederick. The fruit malate contents of the 151 individuals varied greatly, ranging from 1.76 to 11.23 mg/g. The average malate content in blood-fleshed individuals was significantly higher than in non-blood-fleshed individuals (Fig. 3E and Additional file 1: Table S14), consistent with the typically sour taste of blood-fleshed peaches.

**Fig. 4:**
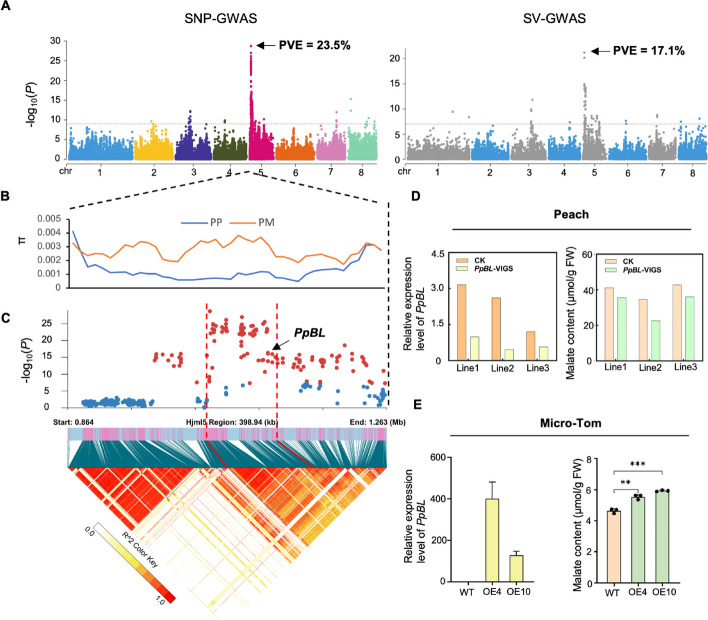
*PpBL* is involved in regulating malate accumulation in peach. **A** Manhattan plot of SNP- (left) and SV-based GWAS (right) of the malate content trait in 301 peach accessions. PVE (phenotypic variance explained) values for the lead SNP and SV at the major locus are indicated. Red dashed horizontal line indicates the significance threshold of GWAS at *p* value of 1e − 9 for SNP-GWAS and 1e − 7 for SV-GWAS, respectively. **B** Nucleotide diversity (π) along chromosome 5 from 864,003 to 1,264,003. Blue line indicates *P. persica*, and yellow line indicates *P. mira*. **C** Significantly associated haplotype block of the major GWAS locus for malate content. The upper panel is a zoom-in view of the Manhattan plot. Red dots indicate SNPs with significant p values. The bottom panel shows the LD plot from Chr5:864,003–1,264,003. **D** Virus-induced gene silencing of *PpBL* in the peach cultivar “Tian Jin Shui Mi.” The relative expression levels of *PpBL* (left) and the malate content (right) in VIGS and control lines are shown. **E** Ectopic expression of *PpBL* in tomato. The relative expression levels of *PpBL* (left) and the malate content (right) in the two OE lines and the control are shown

We then performed GWAS analysis of the malate content trait in the panel of 301 peach accessions. The fruit malate content in these accessions, measured in 2018 and 2019, varied widely, ranging from 0.92 to 12.05 mg/g, and showed a strong correlation between the two seasons (*r* = 0.8033) (Additional file 1: Table S9). We conducted SNP- and SV-based GWAS, both of which identified a major locus (Chr5:1,039,209–1,136,270) associated with malate content (Fig. 4A). This locus overlaps with the previously reported QTL (*D* locus) [44]. The peak SNP (*P* = 1.92 × 10^−29^) and peak SV (*P* = 5.92 × 10^−22^) within this major locus could explain up to 23.5% and 17.1%, respectively, of the phenotypic variation for malate content in our panel.

We calculated nucleotide diversity along chromosome 5 using genome sequencing data from the 301 cultivated peaches (*P. persica*) and 38 wild peaches (*P. mira*; Additional file 1: Table S15). The nucleotide diversity of the major locus and its surrounding region was substantially lower in cultivated peaches than in wild peaches (Fig. 4B), indicating that this locus has likely undergone selection during peach domestication. This major locus contains 11 protein-coding genes, including the *PpBL* gene (Fig. 4C). Of these 11 genes, only *PpBL* (1) showed significantly different expression levels between blood-fleshed and non-blood-fleshed individuals in the F1 population (Fig. 3C), (2) had expression levels strongly correlated with malate content (*r* = 0.7599; Additional file 1: Table S14), and (3) harbored SVs or SNPs in the promoter or coding regions. Transient silencing of *PpBL* in the blood-fleshed peach cultivar “Tian Jin Shui Mi” through virus-induced gene silencing (VIGS) resulted in a significant reduction of malate content in mesocarp tissues compared to the control injected with the empty vector (Fig. 4D). To further verify the functional role of *PpBL* in regulating fruit malate content, we ectopically expressed the gene in tomato cv. Micro-Tom. The malate content in the overexpression (OE) tomato lines was significantly higher than in the wild type (Fig. 4E). Taken together, these results support that the *PpBL* gene, located in the previously reported organic acid locus* D* [44], is likely a key gene involved in regulating fruit malate accumulation in peach.

### PpBL works synergistically with PpNAC1 to activate *PpALMT4* expression

**Fig. 5:**
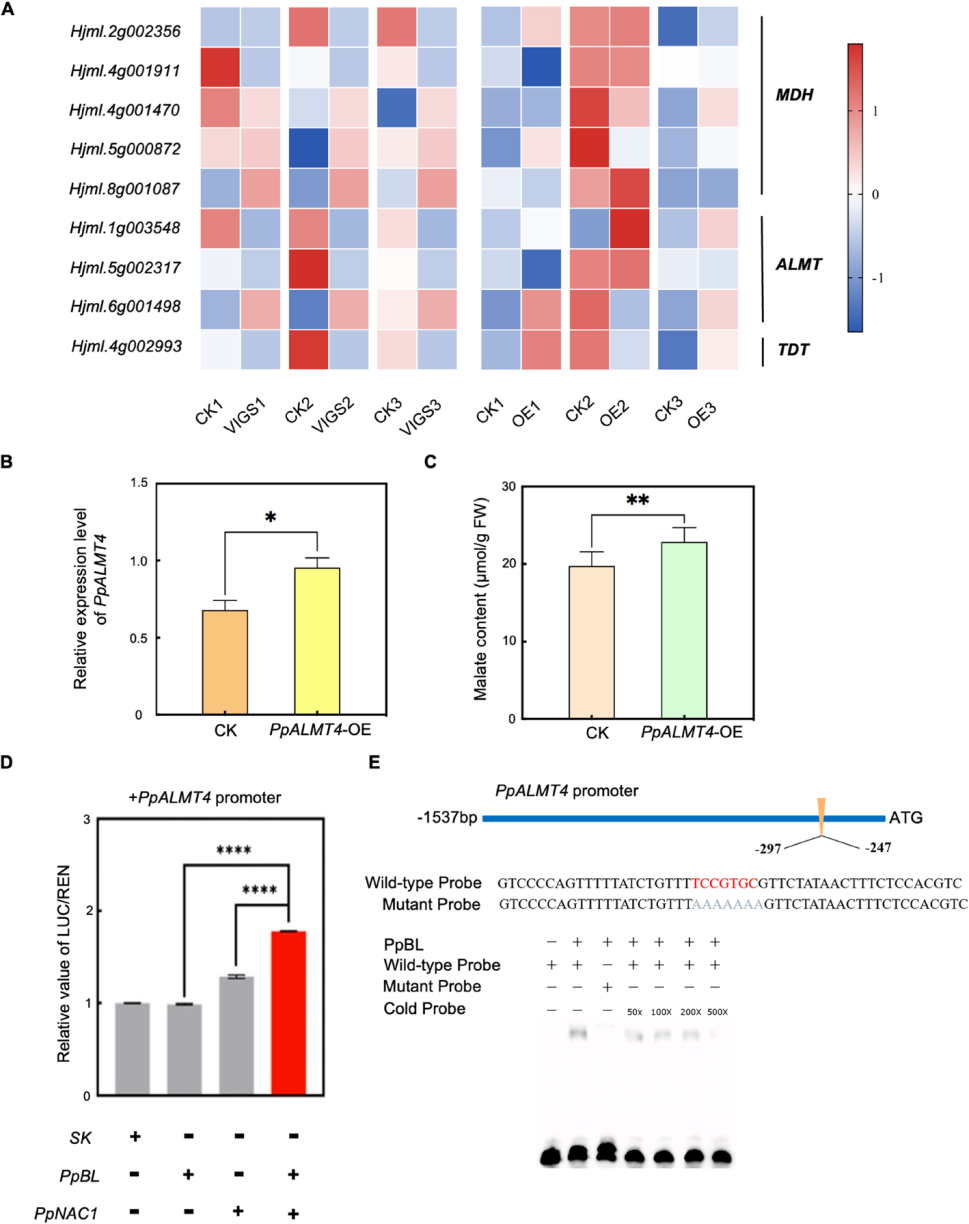
PpBL promotes the expression of *PpALMT4* together with PpNAC1. **A** Expression of *MDH*, *ALMT*, and *TDT* genes in *PpBL*-VIGS and OE lines. **B** Relative expression levels of *PpALMT4* in OE and control (CK) plants. **C** Malate contents in fruit mesocarp of OE and control plants. Fruit mesocarp transiently transformed with the empty vector was used as the control. Data are presented as mean ± SD. Statistical significance was determined using two-sided Student’s paired *t*-tests. **D** Dual-luciferase assays of the transactivation of the *PpALMT4* promoter by PpBL and PpNAC1. The LUC/REN ratio of the empty vector plus promoter (SK) was used as the calibrator (set at a value of 1). Data are presented as mean ± SE from three biological replicates. **** indicate significant differences at *P* < 0.0001 (two-tailed Student’s *t*-tests). **E** EMSA of 3′ boitin-labeled dsDNA probes with the PpBL-binding protein. The mutant bases in the wild-type probe are marked in red, corresponding to those marked in gray in the mutant probe. Presence or absence of the protein or specific probes is marked with symbols + or − . The cold probe concentration was 50 to 500 times that of the wild-type probe

To investigate the molecular mechanisms by which *PpBL* promotes malate accumulation in peach mesocarp, we examined the transcript levels of genes related to organic acid synthesis, degradation, and transport, including those encoding MDHs (malate dehydrogenases), ALMTs, and TDT (tonoplast dicarboxylate transporter) through quantitative PCR (qPCR) analysis. The results showed that transcript levels of *PpALMT4* (*Hjml.1g003548*) were lower in all three *PpBL*-silenced lines compared to their corresponding controls (Fig. 5A). We also performed a transient overexpression assay of *PpBL* in cv. “Xiaohonghua,” a cultivar that barely expresses *PpBL* (Fig. 5A). The results indicated that the expression of *PpALMT4* in the mesocarp of *PpBL-OE* lines was higher than in the control lines. Furthermore, we transiently expressed *PpALMT4* in cv. “Tropic Prince,” a yellow-fleshed peach cultivar, which resulted in a significant increase in *PpALMT4* expression in the mesocarp compared to control lines (Fig. 5B). The malate content was also higher in the overexpression lines (Fig. 5C). To determine whether *PpALMT4* is a direct downstream target gene of PpBL, we performed a dual-luciferase assay. The results indicated that while PpBL alone could not activate the *PpALMT4* promoter, it exhibited a synergistic activation effect on the *PpALMT4* promoter when co-expressed with PpNAC1 (Fig. 5D). Additionally, electrophoretic mobility shift assay (EMSA) demonstrated that PpBL could bind to the *PpALMT4* promoter and activate its transcription when interacting with PpNAC1 (Fig. 5D and E, Additional file 2: Fig. S10).

## Discussion

In this study, we developed high-quality genome assemblies for two peach cultivars, HJML and FHYL, by integrating PacBio HiFi long reads, Illumina paired-end short reads, and Hi-C data, adding valuable resources for peach research and breeding. While single reference genomes have played a pivotal role in advancing peach research and breeding, they cannot capture the full genetic diversity within the peach species and are therefore insufficient for subsequent functional genomics research and molecular breeding. A pangenome that integrates information from multiple genomes representing different ecotypes is urgently needed to enhance our understanding of genome structural variations and the genetic basis of morphotype differentiation in peach. In this study, we constructed a graph pangenome for cultivated peach from sixteen individual peach genome assemblies. The graph pangenome contains 82.3 Mb of sequences that are absent in the reference HJML genome and captures abundant variations among these genomes. Furthermore, graph pangenome analysis identified a retrotransposon insertion in the promoter of the NAC transcription factor *PpBL*, and this insertion is present only in blood-fleshed peaches and enhances *PpBL* expression. *PpBL* has been reported to positively regulate anthocyanin accumulation by activating the expression of *PpMYB10.1* in peach [15]. Based on our data, we hypothesize that the retrotransposon insertion disrupts the binding of putative repressors to the *PpBL* promoter region, located between 654 and 1500 bp upstream of its start codon, thereby upregulating its expression.

The color of fruit flesh and the stability of anthocyanins are influenced by factors such as pH, light, temperature, and structure [45]. At an acidic pH, anthocyanins appear red, while they shift to blue in a basic environment. In apple, the accumulation of both anthocyanins and malate is regulated by *MdMYB1*, which upregulates genes involved in facilitating their transport into vacuoles [46]. In citrus fruits, citric acid and anthocyanin contents appear to have been co-selected during domestication. In pummelo, the accumulation of citric acid and anthocyanins is associated with the high expression of *CgANTHOCYANIN1* (*CgAN1*), and two different MYB transcription factors, *CgPH4* and *CgRuby1* [47]. The *Noemi* gene, which encodes a basic helix-loop-helix (bHLH) transcription factor, has been reported to control the production of flavonoid pigments and fruit acidity in citrus [48]. In our study, GWAS analysis of malate content in 301 cultivated peach accessions revealed a significant locus associated with this trait. This major locus on chromosome 5 contains the *PpBL* gene, which acts synergistically with PpNAC1. Gene silencing and transient overexpression assays confirmed that PpBL promotes malate accumulation in peach fruit by upregulating the expression of the aluminum-activated malate transporter gene, *PpALMT4*. A recent study also identified the retrotransposon insertion in the *PpBL* promoter and associated this insertion with fruit maturity date advancement [49]. Together, these findings highlight the diverse functional roles of *PpBL* in various peach biological processes.

It is worth noting that some non-blood-fleshed cultivars in Group I exhibited high fruit malic acid contents (Additional file 1: Table S9), indicating that malate accumulation in these accessions is regulated by factors other than *PpBL*. The regulatory mechanisms of fruit organic acids are complex. A previous study using multi-omics analysis identified the gene *PpTST1*^*His*^, which encodes a tonoplast sugar transporter, as a regulator of fruit organic acid content in peach. Transient overexpression of *PpTST1*^*His*^ in peach fruits reduced both malate and citric acid contents while increasing total sugar content [21]. In another study, overexpression of the gene *Prupe.5G006500*, which encodes a V-type proton pump F subunit, in peach fruit increased both malate and citric acid contents [22]. Genome resequencing analysis comparing Eastern and Western cultivated peaches revealed *PpALMT1* as a key gene responsible for fruit acidity. This gene encodes an aluminum-activated malate transporter protein, and its overexpression in fruit increases malate content [20]. Notably, both *PpTST1* (Chr5:1,137,757–1,143,153) and *Prupe.5G006500* (Chr5:1,147,448–1,149,852) are located close to the major locus (Chr5:1,039,209–1,136,270) identified in our GWAS analysis (Fig. 4A), while *PpALMT1* is located on chromosome 6 and also affects fruit acidity. These findings suggest that the organic acid content of peach fruit is not controlled by a single locus or gene, and its regulatory network is complex [20–22].

## Conclusions

Overall, we demonstrate that *PpBL* is a key gene in determining malate accumulation in peach, with its expression enhanced by a 6.7-kb retrotransposon insertion in the promoter region. The findings of this study provide valuable insights into the genetic basis of important traits in peach and lay the foundation for future research and breeding efforts in this economically important fruit crop.

## Methods

### Plant materials and organic acid measurement

Young fresh leaves were collected from the peach cultivars HJML and FHYL, the 301 peach accessions used for genome resequencing and GWAS, and the 151 progenies of the F_1_ population along with their two parents (cv. C25-12–11 and cv. Frederick), all grown at the Nanjing National Peach Germplasm Repository in China. Malate content was determined in mesocarp of ripe fruits collected from the 301 peach accessions in 2018 and 2019, and in the peach VIGS lines, as well as in fruits of tomato OE lines at the red ripe stage (7 days after the breaker stage). Malate content determination process included organic acid extraction, vacuum drying, derivatization, compositional analysis by gas chromatography-mass spectrometry (GC–MS), and acquisition of absolute amounts of organic acids, following the protocol described in Fang et al. [50]. Each experiment was performed with three biological replicates.

### DNA and RNA extraction and sequencing

Genomic DNA was isolated from the leaf tissues of HJML, FHYL, and 301 peach accessions using the cetyltrimethylammonium bromide (CTAB) method. High-quality DNA from HJML and FHYL was fragmented using g-TUBE (Covaris) and used to construct a SMRTbell library with the SMRTbell Template Prep Kit (PacBio). The SMRTbell library was sequenced on the Sequel II platform using the CCS mode. For short-read sequencing, one paired-end library was constructed for each of the 301 accessions using the Illumina TruSeq DNA sample preparation kit (Illumina, San Diego, CA, USA) following the manufacturer's instructions, and sequenced on the Illumina NovaSeq 6000 platform with a read length of 150 bp. For Hi-C sequencing, 6 g of HJML young leaves were used for chromatin isolation and Hi-C library preparation using the Proximo Hi-C Plant Kit (Phase Genomics, Seattle, WA, USA), and the library was sequenced on the Illumina NovaSeq 6000 platform with a read length of 150 bp.

For RNA sequencing of the F_1_ population and the fruit and leaf tissues of HJML and FHYL, total RNA was extracted from the flesh of ripe peach fruits and young leaves using the CTAB-based method following the protocol described in Chang et al. [51]. Three biological replicates were conducted for each sequenced individual in the *F*_1_ population. RNA-Seq libraries were constructed for each sample as previously described [52] and sequenced on the Illumina Novaseq 6000 platform.

### Genome assembly

PacBio HiFi reads and Hi-C data were used for genome assembly using hifiasm v0.19.6 [53] with the Hi-C mode. The assembled contigs were then compared against the NCBI non-redundant nucleotide database using BLASTN to identify and remove potential contaminants. To eliminate redundant sequences caused by heterozygosity, the contigs were aligned against themselves, and those with 90% of their length covered by longer contigs were considered redundant and removed. To correct possible sequence errors in the contigs, Illumina reads were mapped to the contigs using BWA v0.7.17 [54], and the contigs were then polished using Pilon v1.23 [55]. Pseudo-chromosomes were constructed using Hi-C data with the 3D-DNA pipeline [56]. Hi-C reads were aligned to the polished contigs using the Juicer pipeline [57].

### Annotation of repetitive elements

MITE and LTR repeat sequences in the HJML and FHYL genomes were identified using MITE-Hunter v8.28 [58] and LTR-Retriever v2.9.0 [59], respectively. RepeatModeler v2.0.1 (http://www.repeatmasker.org/RepeatModeler) was then used to construct a de novo repeat library. MITEs, LTRs, and the de novo repeat library were merged to obtain a combined repeat library. Sequences in the combined repeat library were compared against the SwissProt plant protein database, and those that were likely protein-coding genes were removed. Repeat sequences in the HJML and FHYL genome assemblies were identified by scanning the assemblies with the final repeat library with RepeatMasker (http://www.repeatmasker.org/) and the RepeatRunner subroutine (http://www.yandell-lab.org/software/repeatrunner.html) in the MAKER annotation pipeline [60].

### Gene prediction and annotation

Protein-coding genes were predicted from the HJML and FHYL genome assemblies using MAKER [60]. The transcript evidence included RNA-Seq data from leaf and fruit tissues, as well as RNA-Seq data from leaf, phloem, flower, seed, and root tissues downloaded from the NCBI SRA database (Additional file 1: Table S16). The protein evidence included the complete proteomes of *P. persica* cv. 124 pan, cv. CN14, cv. Lovell, and cv. ChineseCling. All of these sequences were aligned to the genome assemblies using Spaln [61]. MAKER was used to run a set of trained gene predictors, including AUGUSTUS [62], BRAKER2 [63], and GeneMark-ET [64], which were then integrated with transcript and protein evidence to produce the final evidence-based predictions of protein-coding genes. To functionally annotate the predicted genes, their protein sequences were compared against various protein databases, including UniProt (TrEMBL/SwissProt), apple proteomes, tomato proteomes, and four *P. persica* proteomes (cv. 124 pan, cv. CN14, cv. Lovell, and cv. ChineseCling) using BLASTP with an e-value cutoff of 1e − 4.

### Pangenome construction and SV genotyping

The graph-based pangenome for cultivated peach was constructed from 16 genome assemblies [20, 23, 49, 65–72] (**A**dditional file 1: Table S5) using the Minigraph-Cactus pipeline v2.8.2 [41], with the HJML genome as the reference. A gene-based pangenome of the 16 genomes was also constructed based on orthologous groups identified using OrthoFinder [73]. Short reads from the 301 peach accessions were used to genotype variants captured in the graph pangenome using PanGenie [42]. Only SVs (> 50 bp) were retained for downstream analyses.

### Variant calling and population genetic analyses

The cleaned reads from the 301 peach accessions were aligned to the HJML genome using BWA v0.7.17 [54], and only uniquely mapped reads were used for SNP calling using GATK [74]. The resulting raw SNPs were filtered according to the following criteria: (1) SNPs with missing data rate > 20% or minor allele frequency (MAF) < 0.05 were removed; (2) SNPs with genotype quality (GQ) < 30 or located within 5 bp of another SNP were excluded. STRUCTURE (v2.3.4) [75] was used to perform model-based clustering to infer population structure. Twenty independent runs were performed for each *K* value ranging from 2 to 9, with a burn-in length of 10,000 followed by 10,000 iterations, where *K* is the assumed number of populations. The best *K* was determined from the distribution of *ΔK*. The resulting optimal *K* was used in a final run with 100,000 burn-in and 100,000 iterations. Principal component analysis (PCA) was performed using PLINK (v1.9) [76]. Nucleotide diversity (π) was calculated using VCFtools (v0.1.13) [77] on 50-kb sliding windows with a step size of 10 kb across the genome. SNPs located within 400 kb of the peak SNP were used to calculate the *R*^2^ of linkage disequilibrium using LDBlockShow (v1.40) [78].

### GWAS of malate content

The final filtered SNPs and SVs were used separately for GWAS analysis of malate content. To combine malate content data from 2018 and 2019, simulated phenotype data were analyzed by fitting a linear mixed model and calculating the Best Linear Unbiased Predictors (BLUPs) for malate content using the lme4 R package [79]. GWAS was performed using the general linear model in the TASSEL5 package [80]. The Bonferroni-corrected *P* value threshold for significance was estimated as 0.05/n, where n corresponds to the number of SNPs or SVs. The phenotypic variation explained by a marker (*R*^2^) was calculated using TASSEL5 [80].

### RNA-Seq data analysis

Raw RNA-Seq reads were processed to remove adapter and low-quality sequences using Trimmomatic [81]. The resulting reads were then aligned to the HJML genome using HISAT2 [82]. To assist gene prediction, the aligned reads were assembled into transcripts using StringTie [83]. For gene expression analysis, raw counts were normalized to FPKM (fragments per kilobase per million mapped fragments).

### Transient overexpression assay in peach fruit

The coding sequence of *PpBL* was cloned into the pGreenII 0029 62-SK vector for transient overexpression in peach fruit [84]. The recombinant construct and the empty vector control were then transformed into *Agrobacterium tumefaciens* GV3101 (pSoup). The *A. tumefaciens* cultures were adjusted to an OD_600_ of 0.8–1.0 with the infiltration buffer (pH 5.6, 10 mM MES, 150 mM acetosyringone, 10 mM MgCl_2_). *A. tumefaciens* carrying the *PpBL* construct and the empty vector were injected into the left and right sides of the ventral suture of the same peach fruits, respectively, allowing each fruit to served as a paired biological replicate (OE on the left side and control on the right side). The injected peaches were placed in a growth chamber at 20 °C in the dark for 48 h, and then placed in a growth chamber under conditions of 20 °C with a 16-h light and 8-h dark cycle for 5 days. The infiltrated flesh samples were then used for phenotypic and gene expression analyses.

### TRV-based virus-induced gene silencing in peach

The specific *PpBL* coding sequences were amplified and introduced into the pTRV2 vector. Pelleted activated *Agrobacterium* cells carrying either the pTRV1 and pTRV2 construct or the pTRV2-PpBL construct were scraped into the infiltration buffer (pH 5.6, 10 mM MES, 150 mM acetosyringone, 10 mM MgCl_2_), and the concentration was adjusted to an OD600 of 0.5–0.8. The cells were then collected by centrifugation and resuspended in half the volume of the MES infiltration buffer. *Agrobacterium* carrying the pTRV1 and pTRV2 construct were mixed in a 1:1 ratio and infiltrated using syringes. The injection method, reaction time, and fruit storage conditions were the same as those used in the transient overexpression assay described above.

### Generation of transgenic tomato plants

The full coding sequence of *PpBL* was amplified using cDNA templates from “Tianjinshuimi” fruits at the ripening stage and then introduced into the overexpression vector p004. The recombinant construct was transformed into *Agrobacterium tumefaciens* EHA105 competent cells via electroporation prior to plant transformation. Tomato transformation (cv. Micro-Tom) was conducted according to the protocol described in Wang et al. [85]. Transgenic tomato plants were screened for hygromycin resistance, and the expression of *PpBL* in transgenic tomato plants was determined by qRT-PCR using the *RPL2* gene as the reference. The T1 generation of transgenic and wild-type (WT) tomato plants were grown in a growth chamber at 25 °C with a16-h light/8-h dark cycle. Tomato fruits at the red ripe stage (breaker + 7 days) were collected for phenotypic and gene expression analyses. Three plants from each transgenic line were selected as biological replicates, with five fruits selected for each replicate.

### Dual-luciferase assays

According to the protocol described in Yin et al. [86], full-length cDNA of *PpBL* and *PpNAC1* was cloned into the pGreen II 0029 62-SK vector, and the promoter of *PpALMT1* was cloned into the pGreen II 0800-LUC vector. These constructs were transformed into *Agrobacterium tumefaciens* GV3101::pSoup using the Gene Pulser XcellTM Electroporation System (Bio-Rad, Hercules, CA). Activated *Agrobacterium tumefaciens* containing the promoter of *PpALMT1*, *PpBL*, or *PpNAC1* were scraped into the infiltration buffer (pH 5.6, 10 mM MES, 150 mM acetosyringone, 10 mM MgCl_2_), and the concentration was adjusted to an OD600 of 0.75. The *Agrobacterium* culture mixtures consisted of 1 mL of transcription factor solution and 100 μL of promoter solution for transient expression in 4-week-old *Nicotiana benthamiana* leaves. Enzyme activities of firefly luciferase (LUC) and Renilla luciferase (REN) were assayed using dual-luciferase assay reagents (Promega). The ratio of enzyme activities of LUC and REN was measured using a Modulus Luminometer (Promega, Madison, WI) three days after infiltration. The LUC/REN value for the vector SK carrying *PpNAC1* on the promoter was set to 1 as the control. The control and experimental groups were infiltrated on the left and right sides of the same leaf, respectively, and the relative LUC/REN values were calculated using the paired data from the same leaf. At least three biological replicates were performed for each transcription factor–promoter interaction assay.

### Reverse-transcription quantitative PCR (RT-qPCR) analysis

Total RNA was extracted from peach fruits using a CTAB-based method, following the protocol described in Chang et al. [51]. First-strand cDNA was synthesized from the total RNA using HiScript II Q RT SuperMix (+ gDNA wiper) (Vazyme, China). RT-qPCR was performed using a CFX96 instrument (Bio-Rad, Hercules, CA) and ChamQ Universal SYBR qPCR Master Mix (Vazyme, China). *PpTEF2* (encoding a translation elongation factor; GenBank accession no. JQ732180) was used as the internal control to normalize the expression of target genes [87]. Gene expression levels were analyzed using the 2^−△△CT^ method. Primers used for RT-qPCR analysis are listed in Additional file 1: Table S17.

### Recombinant protein purification and EMSA

The coding sequence of *PpBL* was cloned into the pGEX-4 T-1 vector using the primers listed in Additional file 1: Table S17 and then expressed in *Escherichia coli* BL21. Transformed cells were incubated in two 500-mL vials of LB liquid medium containing the Amp resistance gene until the OD600 reached 0.8 and then incubated at 16 °C for 20 h, induced with 0.5 mM isopropyl β-D-1-thiogalactopyranoside (IPTG). Purification of PpBL-GST was conducted using the GST-tag Protein Purification Kit (Beyotime, China). Double-stranded probes were prepared by annealing separately synthesized single-stranded oligonucleotides labeled with biotin at the 3’ end. The probes used for EMSA are listed in Additional file 1: Table S17. EMSA was performed using the LightShift Chemiluminescent EMSA kit (Thermo Fisher Scientific, USA) according to the manufacturer’s instructions.

## Supplementary Information

Additional file 1. Table S1. Summary statistics of the sequencing data for HJML and FHYL. Table S2. Statistics of the HJML and FHYL genome assemblies. Table S3. Assessments of the HJML and FHYL genome assemblies. Table S4. Summary statistics of repeat sequences. Table S5. Published genomes of *P. persica* used for graph pangenome construction. Table S6. GO enrichment of different categories of genes in the gene-based pangenome. Table S7. Location of SVs in the HJML genome. Table S8. GO enrichment of protein-coding genes with exons overlapping SVs in the cultivated peach pangenome. Table S9. List of peach accessions used for genome resequencing and GWAS. Table S10. List of genes containing SVs with higher reference allele frequencies in peach Group VI, within their upstream 2 kb regions. Table S11. List of genes containing SVs with higher reference allele frequencies in peach Group VI, within their exons. Table S12. List of genes containing SVs with higher reference allele frequencies in peach Group I, within their upstream 2 kb regions. Table S13. List of genes containing SVs with higher reference allele frequencies in peach Group I, within their exons. Table S14. List of 38 wild peachaccessions. Table S15. Expression of PpBL, malate content and flesh color in individuals of the F1 population. Table S16. RNA-Seq data used for gene predictions. Table S17. Primers and other oligonucleotides used in this study.

Additional file 2. Figure S1. Fruit photos of peach cultivars used for graph-based pangenome construction in this study. Figure S2. Gene-based pangenome of peach. Figure S3. Length distribution of SVs in the peach graph pangenome. Figure S4. SV densities along the peach chromosomes. Figure S5. Distribution of *∆K* values with *K* from 2 to 9. Figure S6. Fruit malate content and firmness in different peach groups. Figure S7. LTR insertion in the blood-fleshed peach genome validated by read mapping. Figure S8. F_1_ population derived from the cross between the blood-fleshed peach cultivar C25-12-11 and the yellow-fleshed cultivar Frederick. Figure S9. Malate content at different fruit developmental stages of the white-fleshed peach ‘XHH’ and the blood-fleshed peach ‘TJSM’. 

## Data Availability

Raw sequencing reads generated in this study have been deposited in the National Center for Biotechnology Information BioProject database under accession nos. PRJNA1084889 [88] and PRJNA1085020 [89]. Genome assemblies of HJML and FHYL have been deposited in NCBI under accession no. PRJNA1092396 [90] and PRJNA1144061 [91], respectively. Genome assemblies and annotations of HJML and FHYL are also available at figshare [92]. No other scripts and software were used other than those mentioned in the Methods section.
