# Supplementary material for: Graph pangenome reveals the regulation of malate content in blood-fleshed peach by NAC transcription factors
Source: Genome Biol. 2025 Jan 9;26:7. doi: 10.1186/s13059-024-03470-w (PMC11721062; doi:10.1186/s13059-024-03470-w)
Supplement: Supplementary file 2 — Additional file 2. Figure S1. Fruit photos of peach cultivars used for graph-based pangenome construction in this study. Figure S2. Gene-based pangenome of peach. Figure S3. Length distribution of SVs in the peach graph pangenome. Figure S4. SV densities along the peach chromosomes. Figure S5. Distribution of ∆K values with K from 2 to 9. Figure S6. Fruit malate content and firmness in different peach groups. Figure S7. LTR insertion in the blood-fleshed peach genome validated by read mapping. Figure S8. F1 population derived from the cross between the blood-fleshed peach cultivar C25-12-11 and the yellow-fleshed cultivar Frederick. Figure S9. Malate content at different fruit developmental stages of the white-fleshed peach ‘XHH’ and the blood-fleshed peach ‘TJSM’. [file 13059_2024_3470_MOESM2_ESM.pdf]

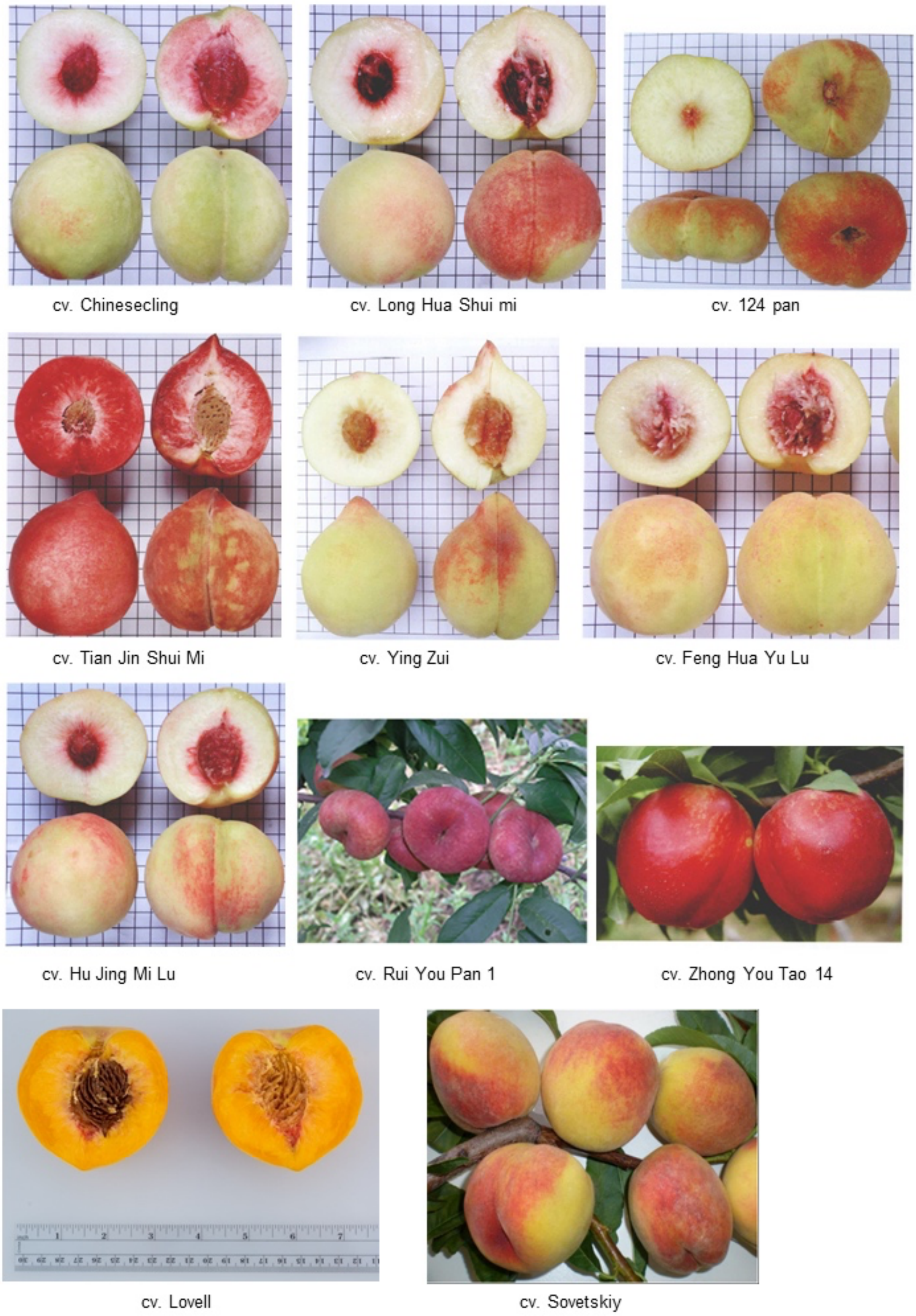

Figure S1. Fruit photos of peach cultivars used for graph-based pangenome construction in this study. Photos were obtained from the following sources: Wang et al., Peach genetic resource in China, ISBN 978-7-109-17303-3; Gladysheva-Azgari et al., PLoS One. 2022, 17:e0269284; Zhou et al., Hortic Res. 2023, 10:uhad210; https://baike.baidu.com/item/%E7%91%9E%E6%B2%B9%E8%9F%A01%E5%8F%B7/6798015; https://fps.ucdavis.edu/treedetails.cfm?v=905

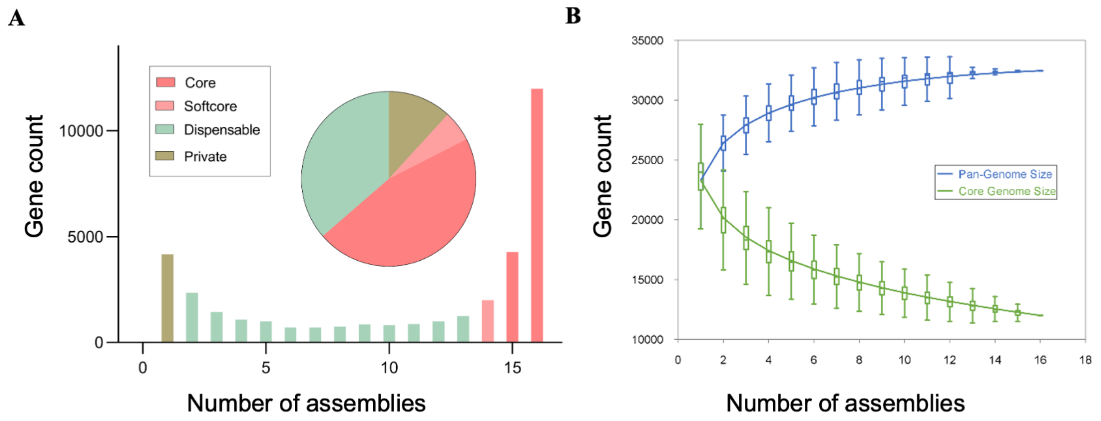

Figure S2. Gene-based pangenome of peach. **A.** Genes shared by different numbers of genome assemblies in the peach pangenome. Core: genes present in 15 or 16 assemblies; Softcore: genes present in 14 assemblies; Private: genes present in one assembly; Dispensable: genes present in 2 to 13 assemblies; **B**. Pangenome and core genome sizes simulated based on gene cluster numbers.

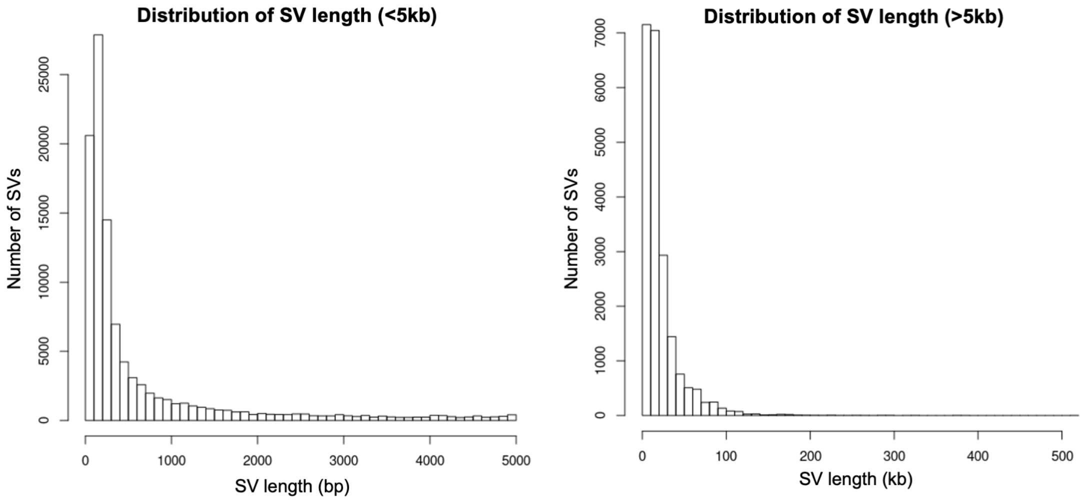

Figure S3. Length distribution of SVs in the peach graph pangenome.

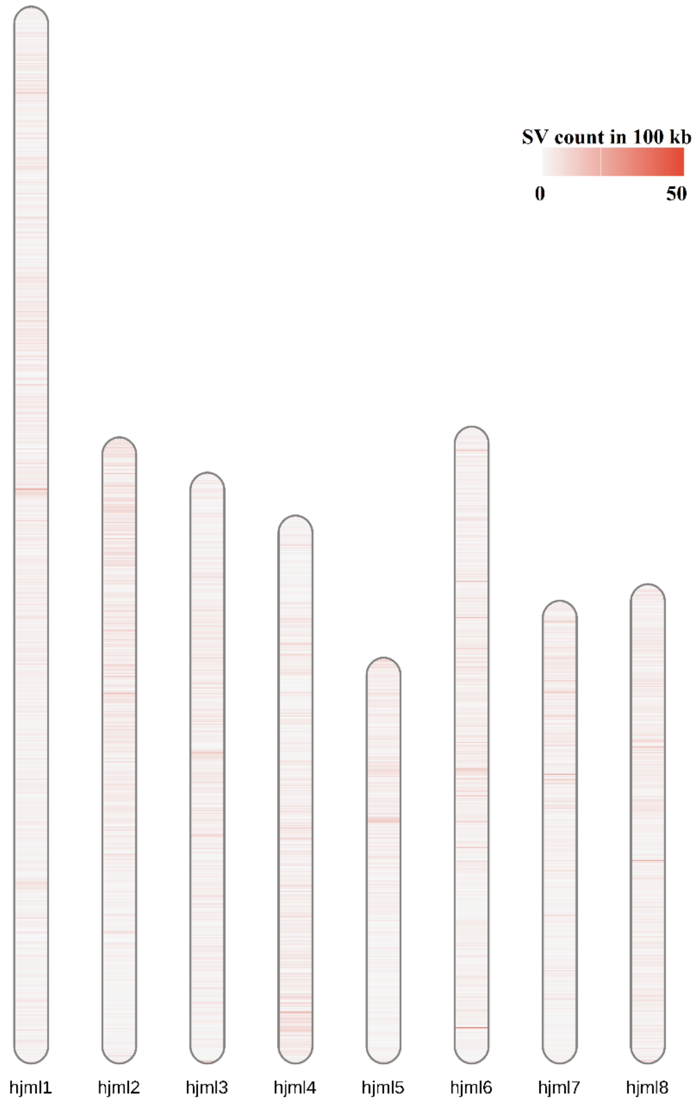

Figure S4. SV densities along the peach chromosomes. Number of SVs in each 100-kb window along the peach chromosomes was calculated.

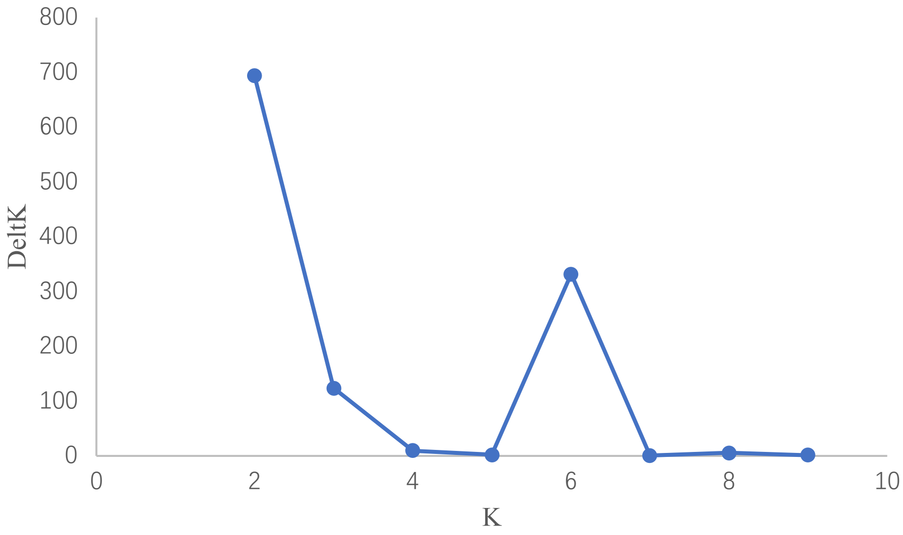

Figure S5. Distribution of Δ*K* values with *K* from 2 to 9.

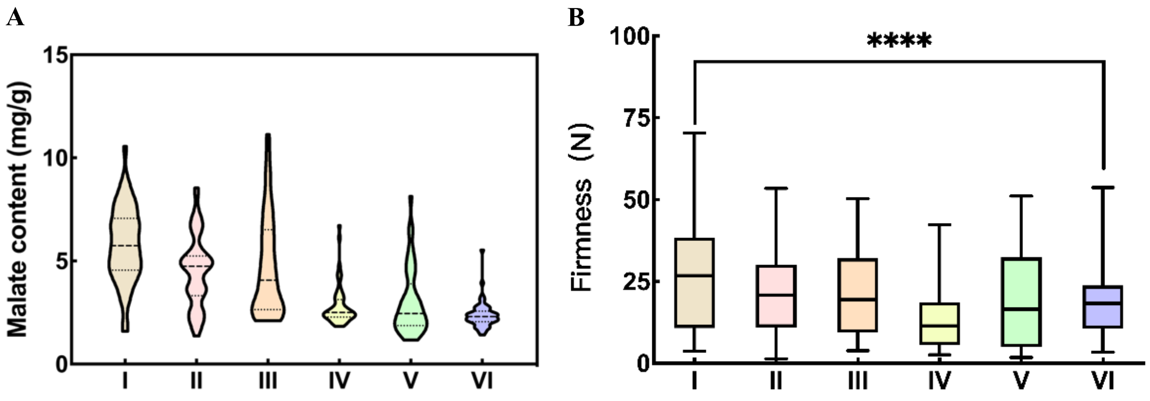

Figure S6. Fruit malate content (**A**) and firmness (**B**) in different peach groups. Asterisks indicate significant differences (∗∗∗∗P<0.0001, two-tailed Student's *t*-test).

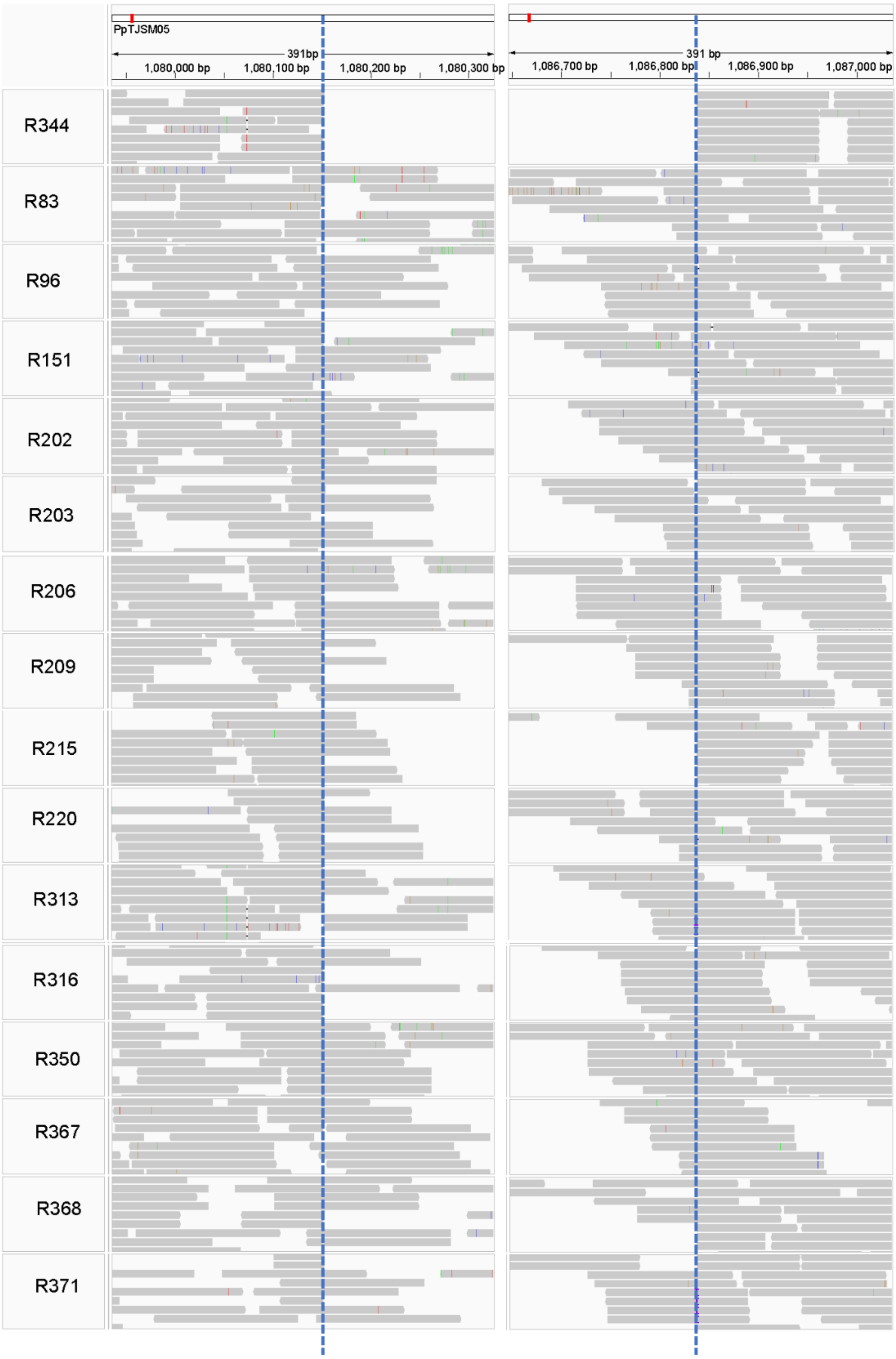

Figure S7. LTR insertion in the blood-fleshed peach genome validated by read mapping. Genome resequencing reads from one white-fleshed and 15 blood-fleshed peaches were mapped to the genome of 'TJSM'. Reads mapped to multiple sites were excluded. The blue dotted lines indicate the 5' and 3' boundaries of the LTR insertion upstream of *PpBL*. The first panel shows read mapping for the white-fleshed peach HJML, while the remaining panels display read mapping for blood-fleshed peaches.

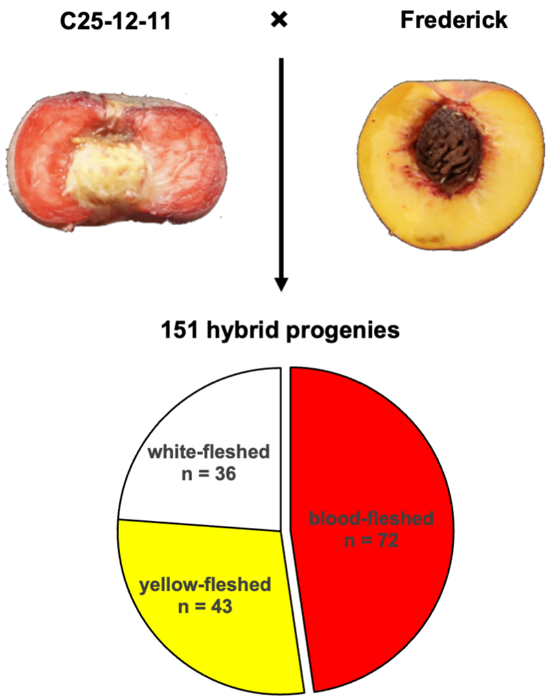

Figure S8. F_1 population derived from the cross between the blood-fleshed peach cultivar C25-12-11 and the yellow-fleshed cultivar Frederick. The population contains 72 blood-fleshed and 79 non-blood-fleshed (43 yellow- and 36 white-fleshed) progenies.

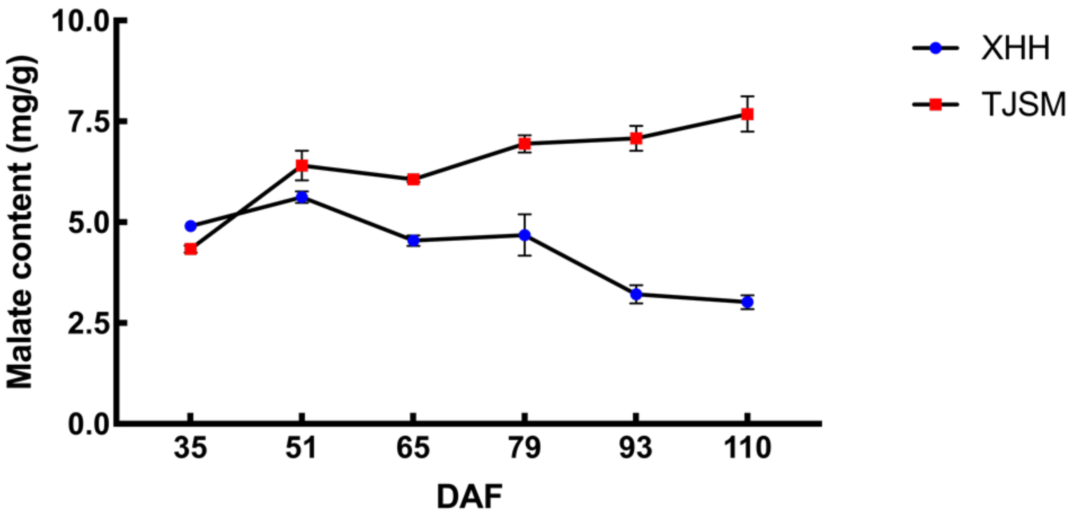

Figure S9. Malate content at different fruit developmental stages of the white-fleshed peach 'XHH' and the blood-fleshed peach 'TJSM'. DAF, days after flowering.

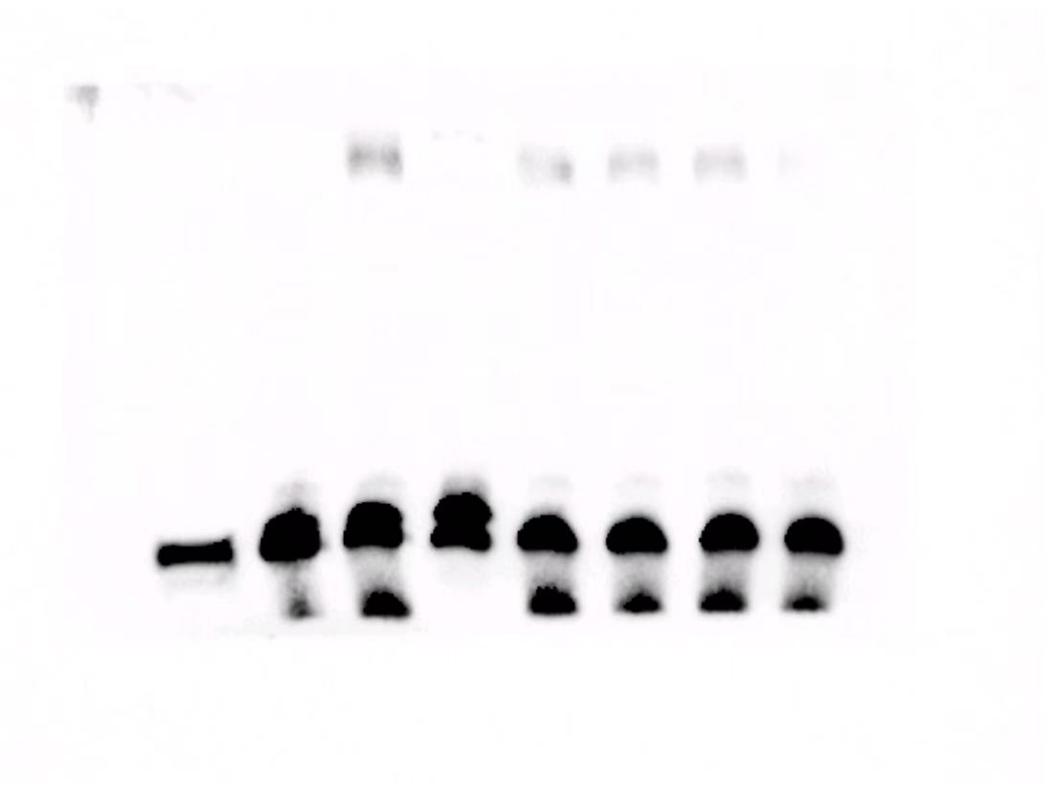

Figure S10. Uncropped image of Figure 5E.
